# From inflammation to carcinogenesis: MiRNA regulates epithelial-mesenchymal transition to facilitate malignant progression of gastric precancerous lesions

**DOI:** 10.3389/fonc.2026.1959240

**Published:** 2026-09-28

**Authors:** Yibing Tian, Tai Zhang, Bochao Yuan, Zhongyu Li, Ting Chen, Yandong Wen

**Affiliations:** 1 Department of Chinese Medicine, Eye Hospital, China Academy of Chinese Medical Sciences, Beijing, China; 2 Department of Gastroenterology, Xiyuan Hospital, China Academy of Chinese Medical Sciences, Beijing, China

**Keywords:** epithelial-mesenchymal transition, gastric precancerous lesions, inflammation, miRNA, non-invasive risk indicators

## Abstract

The process of chronic gastritis transforming into gastric cancer (GC) follows the classic Correa cascade, with persistent inflammation driving the stepwise transition from normal gastric mucosa through superficial gastritis, atrophic gastritis, intestinal metaplasia, dysplasia, and ultimately carcinoma. Among these stages, intestinal metaplasia and dysplasia occurring on an atrophic mucosal background are widely regarded as the most common gastric precancerous lesions (GPL) because of their pronounced carcinogenic risk. Chronic inflammation can induce epithelial–mesenchymal transition (EMT), thereby accelerating gastric malignant progression. In the GPL stage, the inflammatory gastric microenvironment commonly induces partial EMT (pEMT); importantly, this state is partly reversible and may be amenable to intervention to slow disease progression. As lesions advance, epithelial cells more fully undergo EMT, acquiring greater motility, invasiveness, and metastatic capability, which increases susceptibility to malignant conversion. MicroRNAs (miRNAs) are key molecular regulators in the transition from chronic gastritis to GC through EMT modulation. This review comprehensively discusses inflammation-driven EMT activation and functionally classifies pro-EMT oncomiRNAs (e.g., miR-21, miR-155) and anti-EMT tumor-suppressive miRNAs (e.g., miR-200 family, let-7 family, miR-34a), delineating a complex crosstalk network among inflammation, miRNA, and EMT. We further evaluate miRNAs as non-invasive biomarkers for early detection and prognosis of GPL and discuss emerging therapeutic strategies targeting the miRNA–EMT axis, including nanoparticle- or exosome-based delivery systems, miRNA antagonists, and miRNA sponges. Nevertheless, clinical application of circulating miRNAs remains limited by insufficient assay standardization and validation, incomplete target specificity, off-target effects, suboptimal delivery efficiency, and a lack of large-scale clinical evidence. By integrating molecular mechanisms and transformation status of miRNA-EMT axis in GPL, this review provides new insights for precision prevention and targeted intervention in gastric carcinogenesis.

## 1 Introduction

Gastric cancer (GC) ranks as the fifth most prevalent malignancy worldwide and is the fourth leading cause of cancer-related mortality globally (1). Gastric precancerous lesions (GPL) represent a pivotal intermediate stage in gastric carcinogenesis, serving as an important opportunity to interrupt progression and enable early intervention. Chronic inflammation is a principal risk factor in the evolution of GPL to GC; sustained inflammatory injury can drive lesions arising from infectious or autoimmune origins toward malignant transformation (2). This process, often described as “inflammation to carcinoma” transformation, is exemplified by chronic gastritis, a prototypical model of cancer development from persistent inflammation, particularly within the Correa pathway of intestinal-type GC (3). In chronic gastritis, the emergence of intestinal metaplasia and dysplasia (i.e., GPL) against a background of glandular atrophy in the mucosa marks a critical juncture in the continuum from chronic gastritis to GC. Accordingly, effective intervention at the GPL stage is essential for secondary prevention of GC (4).

An imbalance in the gastric immune microenvironment is a critical pathogenic driver of the “inflammation to carcinoma”. Sustained, uncontrolled inflammation can trigger epithelial-mesenchymal transition (EMT) in gastric epithelial cells (5), disrupt the homeostatic balance between cell proliferation and apoptosis, and promote progression from chronic inflammation to GPL and eventually GC (6). A wide range of inflammatory mediators are established as potent EMT inducers (7–9). With persistent inflammatory activation, EMT becomes progressively reinforced, ultimately causing loss of epithelial features, establishment of a tumor-supportive microenvironment, and malignant transformation of epithelial cells (10). Aberrant EMT activation is a pivotal event in the transition from GPL to GC (11), and is governed by multiple interacting signaling pathways (12–14). This activation enhances tumor cell invasiveness and metastatic potential (15), thereby accelerating disease progression along the “inflammation to carcinoma”. MicroRNAs (miRNAs), a predominant class of non-coding RNAs, influence the progression and malignant conversion of GPL by regulating multiple pathways, including those involved in EMT (16), inflammatory responses (17), cell proliferation (18), and apoptosis (19). Thus, elucidating miRNA-mediated regulation during gastric “inflammation to carcinoma” transformation, especially with respect to EMT, is essential for advancing mechanistic understanding of GPL malignancy and for identifying effective diagnostic biomarkers and therapeutic targets.

## 2 Methods

This study employed a narrative review methodology to synthesize literature from the PubMed and Web of Science databases published up to Jul 2026 (**Supplementary Table S1**). The researches were limited to articles published in English. The following combinations of keywords were used: (“MicroRNAs” OR miRNA OR microRNA) AND (“Intestinal Metaplasia” OR “Gastritis, Atrophic” OR “Dysplasia” OR “gastric precancerous lesions” OR “precancerous stomach”) AND (“Inflammation” AND “Carcinogenesis” OR “inflammation-related carcinogenesis” OR “inflammation-driven carcinogenesis”) AND (“Epithelial-Mesenchymal Transition” OR “EMT regulation” OR EMT). Selected studies were analyzed and synthesized to highlight key mechanisms and recent advances. Additional relevant articles were identified through manual screening of reference lists from key reviews and original studies.

## 3 The role of inflammation in gastric malignant progression

Inflammation is a protective host response to a broad range of harmful stimuli, including infection, tissue injury, and chemical irritation. Its principal functions are to eliminate injurious agents and support the repair of damaged tissues. Under normal circumstances, once pro-inflammatory triggers are cleared, the response subsides promptly and reverts to tissue homeostasis, a process often referred to as controllable inflammation. However, when pro-inflammatory stimuli persist or exert prolonged low-grade effects, and the target organ sustains a sustained or excessive inflammatory response, the response may fail to shift from an anti-infective/tissue-damage state to homeostasis, giving rise to uncontrolled inflammation (20). In this state, inflammation persists, driving abnormal cell proliferation and differentiation and thereby promoting the formation of tumorigenic microenvironment (21). This microenvironment can induce genetic mutations and epigenetic changes, ultimately facilitating both tumor initiation and progression (22, 23). During the GPL stage, the gastric mucosa is frequently exposed to long-term injurious stimuli, with Helicobacter pylori (H. pylori) infection being the most prevalent (24), along with injury related to bile reflux (25) and alcohol consumption (26).

### 3.1 Immune cell-mediated modulation of gastric malignant progression

Immune cells, including T lymphocytes, Natural killer (NK) cells, and macrophages, exert complex and context-dependent functions in the gastric inflammation-tumor microenvironment, displaying both tumor-suppressive and tumor-promoting activities depending on their phenotype and the prevailing inflammatory milieu. Tumor-associated macrophages (TAMs), derived from circulating monocytes recruited through chemokines, are particularly representative and can polarize into M1 or M2 phenotypes in response to microenvironmental signals (27). Classically activated M1 macrophages, induced by lipopolysaccharide (LPS), interferon-γ (IFN-γ), and macrophage colony-stimulating factor (M-CSF), produce interleukin-1β (IL-1β) and inducible nitric oxide synthase (iNOS), thereby displaying enhanced antitumor capacity. In contrast, M2 macrophages induced by IL-4, IL-13, or M-CSF secrete IL-10, suppress inflammation, promote extracellular matrix (ECM) remodeling and angiogenesis, and contribute to immune suppression, ultimately facilitating tumor progression (28). M2-polarized macrophages promote gastric malignant progression through regulatory mechanisms mediated by IL-33 and IL-13 (29). In advanced disease stages, TAMs exhibit increased expression of programmed cell death protein 1 (PD-1), allowing PD-L1–high gastric cancer cells to engage this receptor and facilitate immune evasion (27). Concurrently, these macrophages secrete transforming growth factor-β1 (TGF-β1) and IL-10, which suppress CD8+ T-cell function and promote the expansion of regulatory T cells (Tregs) (30).

Tregs constitute another pivotal immune subset that facilitates immune evasion of tumor cells during gastric cancer development (31). They blunt antitumor immunity through multiple mechanisms, including direct suppression of effector T-cell function (32), secretion of immunosuppressive cytokines such as IL-10 and TGF-β, direct cell–cell contact–mediated inhibition of target cells, and depletion of critical growth factors and signaling molecules (e.g., IL-2) (33). CD8+ cytotoxic T lymphocytes (CTLs) are essential mediators of antitumor immunity, exerting direct cytolytic activity against tumor cells through perforin/granzyme-dependent apoptosis and producing effector cytokines, including IFN-γ and Tumor necrosis factor-α (TNF-α). In the immune microenvironment of gastric cancer, however, their function is frequently attenuated or exhausted due to exposure to immunosuppressive factors such as TGF-β and IL-10, as well as metabolic disturbances like dysregulated arginase signaling, thereby promoting tumor progression (27). Th17 cells are also pivotal in the initiation and progression of gastric cancer. Defined by their production of IL-17, these cells can enhance cytotoxic T-cell responses and contribute to anti-tumor immunity under certain conditions (34). However, in the setting of chronic H. pylori-driven inflammation, Th17-derived IL-17 predominantly promotes gastric tumorigenesis by activating NF-κB and STAT3 signaling, accelerating tumor growth and angiogenesis, remodeling the tumor microenvironment (TME), and fostering immune suppression (35). NK cells provide innate immune surveillance through direct lysis of malignant cells, yet their cytotoxic function is substantially attenuated in cancer. Elevated levels of IL-10 and TGF-β1, together with aberrant arginase-mediated metabolism, further inhibit NK-cell effector activity and weaken innate anti-tumor defenses (36, 37). Evidence from the GPL stage indicates that immune dysfunction is already evident within the gastric microenvironment, characterized by impaired macrophage responsiveness, reduced T-cell cytotoxicity, and increased infiltration of functionally exhausted T cells. These findings suggest that disruption of gastric immune homeostasis facilitates malignant progression by progressively eroding anti-tumor immune competence (38).

### 3.2 Inflammatory cytokines-mediated modulation of gastric malignant progression

During GPL progression, immune cells release abundant inflammatory cytokines that induce upregulation of cyclooxygenase (COX) (39) and intestinal mucins such as MUC2 (40), both closely linked to gastric malignant progression. This inflammatory microenvironment can further drive epithelial phenotypic plasticity (41), activate multiple signaling networks (42), and promote the canonical gastric “inflammation to carcinoma” transformation (**Figure 1**).

**Figure 1:**
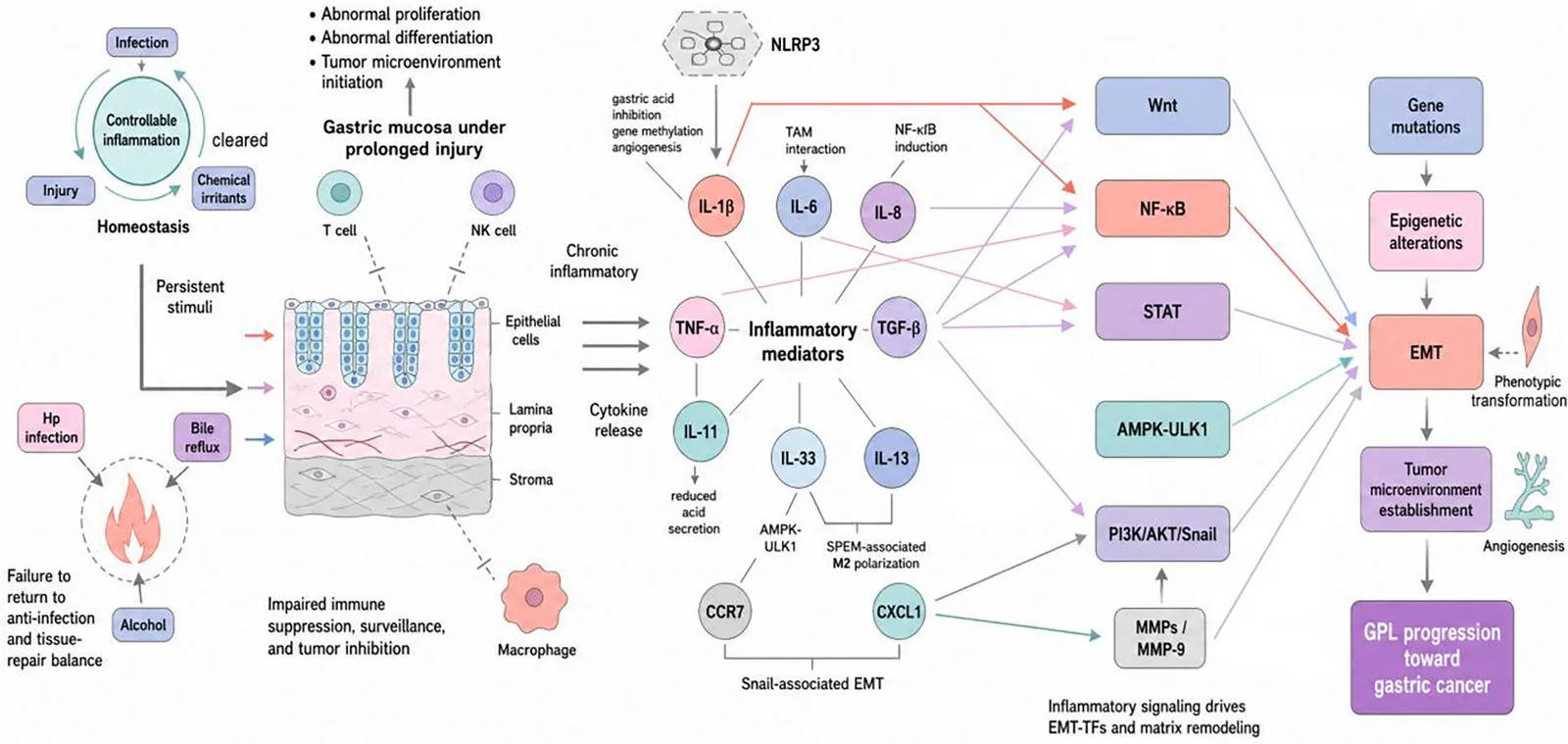
Inflammation enhances EMT and facilitates the conversion of GPL to GC.

ILs play pivotal roles in regulating immune T-cell and B-cell activation and in shaping inflammatory responses. Multiple ILs are critical contributors to the pathobiology of GPL. In particular, IL-1β, IL-8, and IL-17A are significantly elevated in GPL and are central to disease pathogenesis (43). IL-1β is primarily activated by the NLRP3 inflammasome, which governs its release and participates in gastric acid suppression, gene methylation, and angiogenesis (44). It also modulates both Wnt signaling (45) and NF-κB signaling (46), while promoting secretion of intestinal-type mucin MUC13 (47). IL-6 not only facilitates crosstalk between gastric epithelial cells and TAMs (48), but also activates STAT signaling, thereby promoting EMT and accelerating establishment of the gastric TME (8). IL-8 activates NF-κB signaling, induces EMT, and contributes to gastric inflammation-driven malignant transformation (49). IL-11, a parietal cell-derived cytokine, suppresses gastric acid secretion, downregulates parietal cell ion transport genes including CCKb and histamine H2 receptors, and promotes GPL development (50). Moreover, IL-33, a key inflammatory mediator in GPL, acts through the AMPK-ULK1 axis and accelerates GPL progression by promoting GKN1 degradation via the autophagic-lysosomal pathway (51). In addition, IL-33 and IL-13 are markedly upregulated in spasmolytic polypeptide-expressing metaplasia (SPEM), and their effects on gastric metaplasia may be linked to M2 macrophage polarization (29). TNF-α, another central pro-inflammatory cytokine, is substantially elevated in patients with GPL (52); it can activate NF-κB signaling, facilitate EMT, and thereby drive GPL progression (53).

TGF-β belongs to a recently characterized family of cytokines that play essential roles in the regulation of cellular proliferation and differentiation. It is widely regarded as one of the most potent and prevalent inducers of EMT (54) and has been demonstrated to exert a substantial influence on GPL (55). The effects of TGF-β on tumor progression are highly context dependent and exhibit a dual nature (56). Under normal physiological conditions, TGF-β primarily suppresses cellular proliferation and growth. However, during the intermediate and advanced stages of tumorigenesis, this tumor-suppressive activity is progressively attenuated, enabling TGF-β signaling to promote tumor growth and the acquisition of invasive properties. In parallel, TGF-β drives EMT, thereby facilitating stromal deposition and reshaping the tumor immune microenvironment, ultimately accelerating tumor progression (57).

In addition to the aforementioned cytokines, chemokines constitute another crucial class of signaling molecules involved in the “inflammation to carcinoma” transformation. Based on the arrangement of their N-terminal cysteine residues, chemokines are categorized into four major subfamilies: CXC, CC, CX3C, and XC. Beyond their established roles in orchestrating immune and inflammatory responses, chemokines contribute substantially to the formation and remodeling of the TME, including the regulation of EMT and angiogenesis (58). Accumulating evidence indicates that CCR7 and CXCL1 are significantly associated with Snail expression, a key transcriptional inducer of EMT that enhances the migratory and invasive capacities of GC cells (59, 60). Moreover, CCR7 mediates TGF-β1-induced EMT through crosstalk with the NF-κB signaling pathway, thereby facilitating lymph node metastasis and contributing to poorer overall survival in patients with GC (61).

In the context of GPL, dysregulated and persistent inflammation leads to the substantial release of pro-inflammatory mediators, including IL-1β, IL-8, IL-6, TNF-α, and TGF-β. These cytokines regulate EMT-inducing transcription factors (EMT-TFs) through canonical inflammatory signaling pathways, such as Wnt, NF-κB, and STAT, while simultaneously inducing the expression of matrix metalloproteinases (MMPs), thereby facilitating EMT. MMPs constitute a highly conserved family of zinc-dependent endopeptidases that play pivotal roles in the EMT of the gastric malignant progression of GPL (62). In particular, MMP-9 promotes EMT and enhances gastric cancer metastasis by activating the PI3K/AKT/Snail signaling axis (63), and its expression can be further upregulated through the CCR7 or TGF-β1 signaling pathways (64, 65). H. pylori infection contributes to the enhanced expression of MMP family members (66).

### 3.3 Inflammation-induced alterations in miRNA expression and their regulatory role in EMT

During the progression from gastric inflammation to malignancy, inflammation-induced EMT plays a pivotal role in shaping the TME (23). This process also enhances the proliferation, migration, and invasion capabilities of cells, representing a key aspect of GC progression. Inflammatory milieus characterized by the abundant release of pro-inflammatory cytokines can initiate EMT, which, in turn, further promotes the secretion of cytokines such as IL-8, accelerates TME remodeling, and facilitates the activation of cancer-associated fibroblasts (CAFs), collectively contributing to gastric carcinogenesis (67). H. pylori is a significant contributor to this process, which is also influenced by multiple miRNAs. Furthermore, H. pylori promotes CAF formation, thereby inducing localized inflammation and gastric mucosal injury. Through the mediation of multiple signaling pathways, H. pylori enhances the occurrence of EMT and contributes to both the initiation and progression of GPL (68).

Prolonged exposure of the gastric mucosa to inflammatory stimuli, particularly H. pylori infection, leads to the persistent activation of multiple signaling pathways, including TGF-β/Smad, NF-κB, Wnt/β-catenin, and Notch, driven by pro-inflammatory cytokines, reactive oxygen species, and CAFs (**Table 1**). This persistent signaling activation promotes the upregulation of EMT-associated miRNAs, notably miR-21 and miR-155 (69–71). MiR-21 is a pivotal EMT-promoting oncomiR that drives gastric malignant transformation. Its expression is significantly elevated in gastric tissues with high STAT3 levels, and it directly targets tumor suppressors such as PTEN and PDCD4 (72). By suppressing SMAD7, MiR-21 activates TGF-β signaling, promotes epithelial-to-mesenchymal transition, and induces downregulation of epithelial markers accompanied by upregulation of stromal (mesenchymal) markers (73). MiR-155 is another critical oncomiR upregulated during gastritis; it directly targets SOCS1, thereby accelerating gastric mucosal injury and inflammation by enhancing LPS-induced cytokine secretion and NF-κB signaling (74). Conversely, several miRNAs with established EMT-suppressive functions are downregulated under inflammatory conditions (75). For example, during H. pylori infection, miR-375-3p competes for interaction with the 3′ untranslated region (3′UTR) of AEG-1, thereby activating the JAK2/STAT3 signaling pathway and promoting cell proliferation, migration, invasion, and EMT (76). Furthermore, the regulation of EMT by inflammation-induced miRNAs perpetuates a state of gastric inflammation via a positive feedback loop, resulting in a detrimental cycle of “inflammation-EMT-inflammation.” In particular, H. pylori-mediated downregulation of miR-26b-5p increases activity of the PDE4B/CDK8–STAT3 axis, whereas activated STAT3 further suppresses miR-26b-5p transcription, thereby reinforcing this feedback loop and accelerating inflammation and disease progression (17). Likewise, the miR-200 family is downregulated in GPL, and its loss induces a senescence-associated secretory phenotype (SASP) through the TGF-β and TNF-α signaling pathways. This process not only maintains the local proinflammatory microenvironment but also facilitates EMT and extracellular matrix remodeling in neighboring epithelial cells through paracrine signaling (77). Collectively, these regulatory networks highlight the central role of miRNAs in sustaining chronic inflammation in GPL, driving epithelial phenotypic remodeling, and promoting tumorigenesis. Accordingly, the miRNA-mediated inflammation–EMT regulatory network represents a promising therapeutic target for disrupting inflammation-driven GC development and progression.

**Table 1:** Mechanistic summary of miRNA-mediated regulation of EMT in gastric malignant progression.

miRNA	Inflammatory stimulus	Direct target	Signaling pathway	EMT effect	Disease stage	Evidence model	Reference
miR-155	Ethanol, TNF-α, IL-6	SOCS1、GATA3、TP53INP1	NF-κB	Increasing	gastritis, GC	Ethanol-induced acute gastritis mouse model, Ges-1and GC cell lines (MGC-803R and MGC-803S)	(71, 74)
miR-200 family	–	–	TGF-β, TNF-α	Decreasing	GC	GC cells, FKO cells	(77)
miR-21	H. pylori, IL-6, IL-11	PTEN、PDCD4、SMAD7	TGF-β/Smad	Increasing	GPL, GC	Human, GC cell lines (AGS and MKN1), Transgenic mice	(72, 73, 161)
miR-26b-5p	H. pylori	PDE4B、CDK8	STAT3	Decreasing	GC	H. pylori strain cell lines, GC cell lines (MGC803, HGC27, BGC823, and SGC7901) and HEK-293 T, Xenograft-bearing mice	(17)
miR-375-3p	H. pylori	JAK2	JAK2/STAT3	Decreasing	GC	H. pylori-infected mouse, Xenograft-bearing mice, Transgenic mice, GC cells	(76)
miR-520f-3p	–	KLF7	NF-κB	Decreasing	GPL	GPL Mice、MC cell	(122)

## 4 Regulatory mechanisms of EMT in the gastric malignant transformation

EMT is a multifaceted and dynamically regulated biological process that plays a pivotal role in the “inflammation to carcinoma” transformation (6). EMT is characterized by the progressive loss of epithelial features, including apicobasal polarity and intercellular adhesion, together with the acquisition of mesenchymal properties, such as enhanced migratory and invasive capacities and increased resistance to apoptosis. This phenotypic reprogramming is accompanied by the downregulation of epithelial markers, including E-cadherin, β-catenin, and zonula occludens-1 (ZO-1), as well as the upregulation of mesenchymal markers, such as Vimentin and N-cadherin (78). Accumulating evidence indicates that EMT promotes cellular stemness, facilitates immune evasion, and contributes to chemoresistance (79). Physiologically, EMT represents a reversible transition in cellular phenotype and is essential for embryogenesis, wound healing, and tissue regeneration. Based on its biological context, EMT is broadly categorized into three subtypes: type I EMT, which is involved in embryonic development and organogenesis; type II EMT, which is associated with inflammation and tissue repair; and type III EMT, which is closely linked to tumor initiation and progression (78). During the “inflammation to carcinoma” transformation, gastric epithelial cells are induced to undergo EMT through the actions of multiple EMT-TFs, including ZEB, Twist, Snail, OVOL, and GRHL family members, under the influence of inflammatory signals and the TMEs (80). Consequently, epithelial polarity is progressively diminished, cellular migratory capacity is enhanced, and the cells gradually acquire a mesenchymal phenotype (6).

EMT should not be conceptualized as a simple binary switch that drives cells from an epithelial to a mesenchymal state; rather, it represents a complex and dynamic continuum encompassing multiple intermediate phenotypic states. During EMT progression, cells may simultaneously retain epithelial features and acquire mesenchymal characteristics, giving rise to transitional states variously termed partial EMT (pEMT) (80), incomplete EMT (81), and hybrid epithelial/mesenchymal (E/M) phenotypes (82). Zhang et al. demonstrated that pEMT cells exhibit increased expression of EMT-associated transcription factors, including Snai1 and ZEB1, together with upregulation of EMT-related genes such as Fibronectin 1 (FN1) and MMP9, while retaining epithelial markers, including E-cadherin and epithelial cell adhesion molecule (EpCAM) (83). Recent studies have demonstrated that the CD44v3^+^ stem cell subpopulation identified in patients with GC exhibits prominent pEMT features. *In vitro* and *in vivo* experiments further indicate that these cells possess enhanced invasive and metastatic capacities, suggesting that pEMT may facilitate the progression of GPL to GC by sustaining stemness and promoting metastatic traits during gastric malignant transformation (84). Cells in the pEMT state exhibit pronounced phenotypic plasticity, and the acquisition of such plasticity is recognized as a hallmark of malignant tumors (85). Moreover, the pEMT phenotype can contribute to resistance to chemotherapy, while signaling pathways associated with this hybrid E/M state may represent valuable prognostic biomarkers and promising therapeutic targets (86). Additionally, the concurrent epithelial adhesive and mesenchymal migratory properties of cells with a hybrid E/M phenotype facilitate collective migration, thereby potentially contributing to malignant progression (87). Notably, accumulating evidence suggests that hybrid E/M states are regulated by miRNAs, EMT-TFs, and other molecular determinants, and may consequently revert to a predominantly epithelial phenotype (88). Therefore, early intervention to inhibit EMT in epithelial cells during the initial stages of disease may be critical for delaying disease onset and impeding subsequent progression.

In the initial phases of GPL, EMT was frequently triggered by chronic inflammation, H. pylori infection, and other contributing factors (89). Recent studies have demonstrated that the H. pylori virulence factor CagA activates the YAP/ZEB1 signaling axis, thereby initiating gastric epithelial cell plasticity. Subsequently, METTL3-mediated m6A RNA modification sustains the mesenchymal phenotype in the absence of ongoing bacterial stimulation. Ultimately, the inflammatory microenvironment established by CAFs and TAMs further stabilizes this hybrid E/M state (i.e., pEMT), through paracrine feedback signaling (90). At this stage, most gastric epithelial cells exhibit pEMT, characterized by reduced E-cadherin expression and increased N-cadherin levels. Therapeutic interventions may reverse the EMT process and thereby impede the progression of GPL (91). Multiple signaling pathways have been implicated in the regulation of this process. Yu et al. reported that, during the CAG stage, activation of the JAK/STAT signaling pathway occurred concurrently with pEMT. Although E-cadherin expression in the CAG model group did not differ significantly from that in the normal control group, the expression of mesenchymal markers, including Vimentin, was markedly increased. The YQHYJD compound effectively suppresses gastric malignant progression by inhibiting pEMT mediated through the JAK2/STAT3 signaling pathway (92). Additionally, Cai et al. demonstrated that activation of the CCL2/CCR2 chemokine signaling axis promotes cell migration and EMT in malignant cells, specifically malignant cells (MC) (i.e., GES-1 malignant transformed cells) (93). Furthermore, during the gastritis stage, H. pylori CagA may stimulate the coordinated biphasic regulation of LATS2 and YAP1, thereby mediating EMT reprogramming through the Hippo/LATS2/YAP1/TEAD signaling pathway (94). In addition, the miR-200/ZEB1 negative-feedback loop and the inflammatory response associated with NF-κB signaling induced by H. pylori also play important roles in the development of gastritis and pEMT (95).

As gastric inflammation progresses toward malignancy, EMT becomes increasingly evident. At the GC stage, tumor cells acquire greater invasive and metastatic potential, accompanied by increasing complexity within the TME and in intercellular interactions. In parallel, gastric epithelial cells undergo profound phenotypic and biological alterations, which are closely associated with poor prognosis in affected patients (96). Accumulating evidence indicates that EMT-driven proliferation and invasion of GC cells are not mediated exclusively by the canonical TGF-β and Wnt/β-catenin signaling pathways, but also arise from the crosstalk among multiple additional signaling cascades (97, 98). For instance, CHD4-mediated activation of the MYH9/GSK3β/β-chain protein axis can stimulate WNT signaling, thereby inducing EMT and promoting the migration, invasion, and metastasis of GC cells (98). Additionally, tumor-associated neutrophils (TAN) (99), Tregs (100), and other cellular components of the TME contribute substantially to the regulation of EMT in GC. Furthermore, GC cells upregulate stemness-associated markers, including CD54, CD44, SOX2, and Lgr5, thereby acquiring enhanced stem cell-like properties. This process not only suppresses apoptosis and increases chemoresistance but also further intensifies the EMT phenotype (101, 102).

In conclusion, EMT associated with GPL is a complex and multifactorial process governed by the coordinated interplay of diverse signaling pathways, intercellular interactions, inflammatory responses, and alterations in the TME (**Figure 2**). With the continued advancement of research on EMT, an expanding body of evidence has clarified the underlying molecular mechanisms and their intricate interactions, particularly the regulatory roles of miRNAs in modulating EMT in gastric epithelial cells. Comprehensive elucidation of this regulatory network will deepen our understanding of the biological basis of the “inflammation to carcinoma” transformation in GPL, facilitate the identification of molecular targets capable of reversing EMT at its early stages, and provide a theoretical foundation for developing novel targeted therapeutic strategies to prevent GC or improve clinical outcomes in patients with GC.

**Figure 2:**
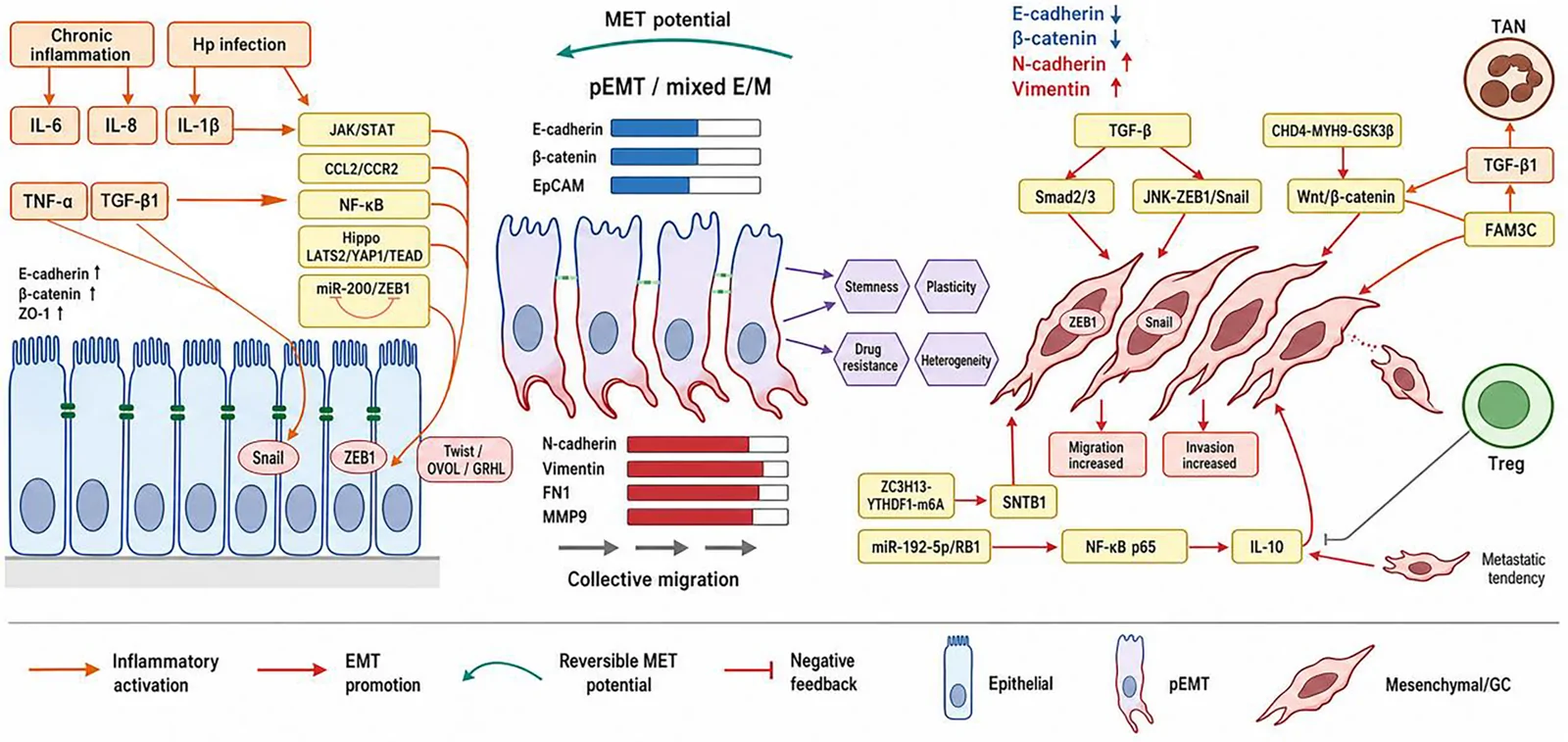
Schematic representation of the process of gastric EMT.

## 5 Regulatory mechanism of miRNA in the EMT process

Recently, the rapid advancement of high-throughput sequencing and multi-omics technologies has facilitated the identification of candidate miRNAs and their associated regulatory networks during the early stages of GC particularly throughout the progression from GPL to invasive adenocarcinoma. Nevertheless, these approaches primarily provide insights into potential regulatory mechanisms and molecular targets. Accordingly, numerous studies have subsequently employed *in vivo* and *in vitro* models to elucidate the specific mechanisms underlying miRNA-mediated regulation. Accumulating evidence suggests that miRNAs exert bidirectional regulatory effects on the EMT of gastric mucosal epithelial cells by targeting core EMT-associated transcription factors, including Snail, Slug, ZEB1/2, and Twist, as well as key signaling pathways such as TGF-β, Wnt/β-catenin, Notch, and PI3K/AKT (**Table 2**) (16, 77). Oncogenic miRNAs (oncomiRs) can promote EMT initiation and thereby facilitate the malignant progression of GPL. Conversely, a subset of tumor-suppressive miRNAs is downregulated, resulting in the loss of negative regulation of EMT transcription factors and their associated signaling cascades, which ultimately accelerates malignant progression (103, 104).

**Table 2:** Summary of miRNAs regulating EMT.

miRNAs	Status	Study types	Signaling pathway/targets	Regulatory molecules	EMT effectson	Reference
let-7a-5p	Downregulated	Human, GC cell lines (NCI-N87、SNU-1、KATO III)	Wnt/β-catenin pathway	TTTY15	Decreasing	(124)
let-7i-3p	Downregulated	GC cell lines (MGC-803, SGC-7901, HGC-27), Xenograft-bearing mice	CCND1/NF-κB pathway	/	Decreasing	(123)
miR-118	Upregulated	Human, GC cell lines (HGC-27, AGS) and MC cells, Xenograft-bearing mice	TRPM2	METTL3	Increasing	(107)
miR-124-3p	Downregulated	AGS cells and HEK-293T cells	ITGB1	/	Decreasing	(170)
miR-125a-5p	Downregulated	GC cell lines (AGS, HGC-27, MKN-45, and SGC-7901), Xenograft-bearing mice	JARID2、PI3K/AKT pathway	lncRNA TNK2 AS1	Decreasing	(132)
miR-125a-5p	Downregulated	Human, GC cell lines (HGC27, MKN-45 and AGS)	SERPINE1	DNMT1	Decreasing	(135)
miR-1276	Downregulated	Human, GC cell lines (MKN-7 and AGS), Xenograft-bearing mice	SLC7A11	circTMEM87A	Decreasing	(149)
miR-128-3p	Downregulated	Human, GC cell lines (MGC-803, BGC-823, and SGC-7901)	TUFT1	/	Decreasing	(120)
miR-1299	Downregulated	Human, GC cell lines (SGC-7901, BGC-823, AGS, HGC-27, and MKN-45)	RHOT1	circ_0005230	Decreasing	(150)
miR-130a-5p	Downregulated	Human, GC cell lines (HGC-27, BGC-823, MKN-74 and AGS)	POU2F1	Linc01748	Decreasing	(171)
miR-133a-5p	Downregulated	Human, GC cell lines (MGC-803, HGC-27, BGC-823, and SGC-7901)	VIM	LINC01503	Decreasing	(147)
miR-142	Downregulated	Human, GC cell lines (AGS, MKN-45, MKN-28, SGC-7901 and BGC-823), Xenograft-bearing mice	LRP8	/	Decreasing	(75)
miR-144	Downregulated	GC cell lines (HGC-27 and BGC-823), Xenograft-bearing mice	mTOR pathway	/	Decreasing	(155)
miR-144-3p	Downregulated	Human, GC cell lines (SGC-7901, BGC-823, MKN-45, HGC-27)	EZH2	circKIF4A	Decreasing	(154)
miR-145-5p	Downregulated	Human, GC cell lines (BGC-823, MGC-803, HGC-27, and SGC-7901), Xenograft-bearing mice	SERPINE1、ERK1/2 pathway	/	Decreasing	(138)
miR-145-5p	Downregulated	GC cell lines (AGS, MKN-45, MKN-7, HGC-27) and HEK-293T, Xenograft-bearing mice	YTHDF1	PLAGL2/UCA1	Decreasing	(139)
miR-149-5p	Downregulated	Human, GC cell lines (MKN- 28, MKN-45, KATO-3, HGC-27, SNU-216 and AGS), Lung Metastasis Model	Men1、HSPA6/JNK/JUND	/	Decreasing	(172)
miR-15a-5p	Downregulated	Human, GC cell line (HGC-27), Xenograft-bearing mice	CCND1	lncRNA TSPEAR-AS2	Decreasing	(102)
miR-15b-5p	Downregulated	Human, MC cells, Xenograft-bearing mice	KIF1B/PI3K/AKT pathway	circ0000549	Decreasing	(178)
miR-16-5p	Downregulated	Human, GC cell lines (MGC-803 and SGC-7901)	YAP1/Hippo pathway	LINC00649	Decreasing	(140)
miR-181-5p	Downregulated	Human, GC cell lines (MKN45, SGC- 7901, MGC-803, and BGC-823), Xenograft-bearing mice	METTL3/KLHL5	/	Decreasing	(117)
miR-181a-5p/miR-181b-5p	Upregulated	Human, GC cell lines (AGS, SNU-216, MKN-45, HGC-27, SNU-5, MKN-74, KATO-3, and NCI-N87), Xenograft-bearing mice	SOCS3/NFATC2、LncRNA-MIR181A1HG/ELAVL1	/	Increasing	(115)
miR-184	Upregulated	Human, GC cell lines (AGS and NCI- N87), Xenograft-bearing mice	FOSB	circ_0021087	Increasing	(106)
miR-192-5p	Upregulated	Human, GC cell lines (MKN45 and BGC-823), Xenograft-bearing mice	RB1/NF-κBp65/IL-10	/	Increasing	(100)
miR-195-5p	Downregulated	GAC cells (SGC-7901 and BGC-823)	OTX1	/	Decreasing	(142)
miR-195-5p	Downregulated	Human, GC cell lines (SGC7901 and MGC803)	PD-L1	/	Decreasing	(143)
miR-196a-5p	Upregulated	GC cell lines (AGS, MKN-45, and HGC-27), Peritoneal Dissemination Model	FBXO45/Snai1	/	Increasing	(16)
miR-199a-3p	Downregulated	GC cell lines (SGC-7901, MGC-803), Xenograft-bearing mice	DDR2-mTOR-SOX2	/	Decreasing	(141)
miR-199b-5p	Upregulated	Human, GC cell lines (AGS, MGC803, SGC7901), Xenograft-bearing mice	HHIP	/	Increasing	(116)
miR-200a	Downregulated	GC cell lines (MKN-45, SNU-1, HGC-27, AGS, and MGC-803), Xenograft-bearing mice	Bcl-2 pathway	lncRNA-ATB	Decreasing	(121)
miR-200 family	Downregulated	AGS cells, FKO cells	TGF-β and TNF-α pathways	/	Decreasing	(77)
miR-203a-3p	Downregulated	Human, GC cell lines (MGC-803, MKN-45, BGC-823, SGC-7901 and HGC-27)	TGF-β、Wnt/β-catenin pathway	lncRNA XLOC_004787	Decreasing	(127)
miR-204-5p	Downregulated	Human, GC cell lines (AGS, SNU-1, NCI-N87), Xenograft-bearing mice	VCAM1	LINC01480	Decreasing	(179)
miR-206	Downregulated	Human, GC cell lines (AGS, KATO III, SNU-5, HGC27 and NCI-N87), Xenograft-bearing mice	ADAM12	lncRNA FAM225A	Decreasing	(153)
miR-20a-5p	Downregulated	Human, GC cell lines (AGS, MKN45, SGC7901, SUN5, and HGC27), Xenograft-bearing mice	E2F1	lncRNA HAGLR	Decreasing	(173)
miR-211-3p	Downregulated	Human, GC cell lines (HGC-27, AGS, MKN74, and GTL-16)	TRIM29	lncRNA ELFN1-AS1	Decreasing	(174)
miR-21-3p	Upregulated	Human, GC cell lines (AGS, HGC-27), Xenograft-bearing mice	SMAD7、TGF-β pathway	circWNK1	Increasing	(73)
miR-215-3p	Downregulated	Human, GC cell lines (HGC-27, KATO III, NCI-N87, and AGS)	CXCR1	lncRNA PCED1B-AS1	Decreasing	(152)
miR-217	Downregulated	Human, GC cell lines (AGS, NCI-N87, SNU-5 and KATO III)	ANLN	circ_0000144	Decreasing	(151)
miR-26-5p	Downregulated	Human, GC cell lines (MGC-803 and AGS)	PFKFB3/TGF-β/SAMD pathway	/	Decreasing	(128)
miR-28-3p	Downregulated	GC cell line (MKN-45), Xenograft-bearing mice	ARF6/Hedgehog pathway	/	Decreasing	(118)
miR-29a/b/c-3p	Downregulated	GC cell lines (HGC27 and SNU-1)	HOXA10、p38 MAPK/STAT3 pathway	lncRNA HOXA10-AS	Decreasing	(133)
miR-29a-3p	Downregulated	Human, GC cell lines (AGS, SGC-7901)	VAMP7	lncRNA NR2F1-AS1	Decreasing	(18)
miR-29c	Downregulated	GC cell lines (SGC7901 and MKN28) and HEK-293T, Xenograft-bearing mice	VEGFA/VEGFR2/ERK	/	Decreasing	(134)
miR-30a-5p	Downregulated	GC cell lines (SGC-7901, MGC-803, HGC-27, AGS)	PHTF2/AKT pathway	/	Decreasing	(130)
miR-320b	Upregulated	Human, GC cell lines (AGS, MGC-803, SGC-7901 and SNU-1)	PDCD4	lncRNA NR2F2-AS1	Increasing	(114)
miR-362-3p	Downregulated	GC cell lines (SGC-7901, HGC-27)	DEP-1/ERK pathway	/	Decreasing	(137)
miR-3681-3p	Downregulated	Human, GC cell lines (AGS, HGC-27, SGC-7901 and MKN45), Xenograft-bearing mice	MAP2K7	lncRNA PCED1B-AS1	Decreasing	(146)
miR-375-3p	Downregulated	H. pylori-infected mouse, Xenograft-bearing mice, Transgenic mice, GC cell line (BGC-823) and HEK-293T	JAK2/STAT3 pathway	AEG-1	Decreasing	(76)
miR-378d	Downregulated	Human, GC cell lines (SGC-7901, MKN-45, HGC-27 and AGS), Peritoneal Metastatic Model	METTL4	/	Decreasing	(186)
miR-384	Downregulated	Human, GC cell lines (AGS, HGC-27, MKN-28, MKN-45), Xenograft-bearing mice	CTNNB1	/	Decreasing	(182)
miR-424-3p	Downregulated	GC cell lines (SGC-7901 and AGS), Xenograft-bearing mice	RHOXF2	/	Decreasing	(180)
miR-424-5p	Upregulated	Human, GC cell lines (AGS, MGC803, BGC-823, SGC-7901, HGC-27), Xenograft-bearing mice	Smad7、TGF-β pathway	lncRNA MBNL1-AS1	Increasing	(109)
miR-4424	Downregulated	Human, GC cell lines (HGC27, MKN28, MKN45, and AGS), Xenograft-bearing mice	OAS2	lncRNA NKILA	Decreasing	(148)
miR-455-3p	Downregulated	Human, GC cell lines (HGC-27 and AGS), Xenograft-bearing mice	ARMC8/Wnt/βcatenin pathway	/	Decreasing	(125)
miR-4731-5p	Downregulated	Human, GC cell lines (AGS, MKN-45, HGC-27, and SGC-7901)	HOXC6	LINC01857	Decreasing	(103)
miR-4742-5p	Upregulated	GC cell lines (MKN1, MKN74, AGS, and NCI-N87)	Rab43、AKT、NF-κB	/	Increasing	(108)
miR-486-5p	Downregulated	GC9811 cells, GC9811-P cells, and HMrSV5 cells	SAMD2、CDK4、ACTR3	/	Decreasing	(177)
miR-490-3p	Downregulated	Human, GC cell lines (MKN-45, AGS, BGC-823, SGC-7901, MGC-803, MKN-74)	HAVCR1、MEK/ERK pathway	lncRNA AL139002.1	Decreasing	(136)
miR-491-5p	Downregulated	Human, GC cell lines (AGS, MGC-803, HGC-27), Xenograft-bearing mice	HMGA2	lncRNA CASC19	Decreasing	(175)
miR-493-3p	Downregulated	Human, GC cell lines (BGC-823, SGC-7901)	XPR1	circRNA_102191	Decreasing	(145)
miR-493-5p	Downregulated	Human, GC cell lines (HGC27, AGS, and NCI-N87)	IRS2	circPRKDC	Decreasing	(144)
miR-501-3p	Upregulated	GC cell lines (SGC-7901, MKN-28, AGS, HGC-27)	TM4SF1	/	Increasing	(19)
miR-5095	Downregulated	Human, GC cell lines (AGS, HGC-27), Xenograft-bearing mice	CEACAM5	/	Decreasing	(183)
miR-511-5p	Downregulated	Human, GC cell lines (AGS, HGC-27, SGC-7901, MKN-28, BGC-823)	PAK2	/	Decreasing	(104)
miR-513b-5p	Upregulated	GC cell lines (AGS, HGC -27) and human monocytes THP-1, Xenograft-bearing mice	PTEN/AKT/mTOR pathway	/	Increasing	(111)
miR-519d-3p	Downregulated	Human, GC cell lines (MKN45 and HGC-27), Xenograft-bearing mice	HMGA2	lncRNA ROR	Decreasing	(184)
miR-520f-3p	Downregulated	GPL Mice、MC cell	KLF7/NFκB pathway	/	Decreasing	(122)
miR-526b-5p	Downregulated	TGES-1	/	TGES-1-EX circ0008274	Decreasing	(181)
miR-574-3p	Upregulated	Human, GC cell line (NCI-N87), Xenograft-bearing mice	CUL2/HIF-1α	/	Increasing	(110)
miR-575	Upregulated	Human, GC cell lines (AGS, MGC803, MKN45, MKN28)	BLID	lncRNA RNU1	Increasing	(112)
miR-635	Downregulated	GC cell lines (AGS, HGC27), Xenograft-bearing mice	HMGA1	/	Decreasing	(169)
miR-642b-3p	Upregulated	Human, GC cell lines (SNU-5, AGS, MKN-74, HGC-27, and KATO III), Xenograft-bearing mice	CSMD1、SAMD pathway	/	Increasing	(105)
miR-665	Downregulated	Human, GC cell lines (AGS, HGC-27, MKN-45, and MKN-74), Xenograft-bearing mice	HMGB1、Wnt/β-catenin pathway	circ_0006646	Decreasing	(126)
miR-6809-3p	Downregulated	Human, GC cell lines (HGC-27, MGC-803, SGC-7901, BGC-823) and HEK-293T, Xenograft-bearing mice	ZNF90/TGF-β1/SMAD2/3 pathway	circMRPL35	Decreasing	(13)
miR-7	Downregulated	GC cell lines (HGC-27, SGC-7901, MGC-803 and BGC-823), Xenograft-bearing mice	FAK/AKT/mTOR pathway	/	Decreasing	(131)
miR-7160	Downregulated	Human, GC cell lines (BGC-823 and HGC-27)	SIX1	/	Decreasing	(119)
miR-876-5p	Upregulated	GC cell lines (SGC-7901, BGC-823, MKN-45, HGC-27, MGC-803, and AGS), Abdominal Metastasis Model	GNG7	circRNA CDR1as	Increasing	(113)

### 5.1 Types of pro-EMT miRNAs and their regulatory pathways

During the progression from gastric inflammation to GPL and GC, a number of oncogenic miRNAs are markedly upregulated and promote EMT and malignant phenotypes by modulating key signaling pathways (105, 106) (**Figure 3**). During the early stages of the “inflammation to carcinoma” transformation in GPL, miRNAs regulate gastric inflammatory responses. Research indicates that miR-155 targets SOCS1, thereby exacerbating gastric mucosal injury and inflammation (74). As gastric epithelial cells undergo malignant transformation, an increasing number of miRNAs have been identified as positive regulators of EMT (19). An investigation utilizing N-methyl-N’-nitro-N-nitrosoguanidine (MNNG) to induce GES-1 cells for the establishment of an MC cell model revealed that miR-118 can facilitate EMT in gastric epithelial cells by targeting TRPM2 (107). Furthermore, miRNA-mediated regulation of EMT is closely linked to inflammation-associated signaling pathways and may further amplify the gastric inflammatory response (108). The TGF-β/Smad pathway, a well-established central regulator of EMT, is modulated by multiple miRNAs. Among these, miR-21-3p and miR-424-5p promote EMT in gastric epithelial cells by targeting SMAD7 and thereby potentiating TGF-β signaling (73, 109). Other miRNAs, including miR-574-3p and miR-513b-5p, further facilitate EMT through the regulation of HIF-1α- and AKT-related signaling pathways (110, 111). MiRNAs play a critical role in EMT in gastric epithelial cells, their expression and activity are also regulated by other ncRNAs, including lncRNAs and circRNAs (112, 113). For instance, lncRNA NR2F2-AS1 acts as a molecular sponge for miR-320b, thereby diminishing its repressive effect on the target gene PDCD4. This interaction consequently suppresses EMT and GC progression, suggesting that NR2F2-AS1 may represent a potential therapeutic target for the prevention and treatment of GC (114). Certain miRNAs may also exert reverse regulatory effects on ncRNA expression, exemplified by miR-181a-5p/miR-181b-5p, which downregulates SOCS3 and enhances NFATC2-mediated transcriptional activation of the MIR181A1HG promoter, creating a positive feedback loop that stabilizes ELAVL1, thereby promoting GC infiltration, metastasis, and EMT (115). The circulating and tissue levels of these miRNAs in patients with GC are frequently associated with disease stage, prognosis, and therapeutic response (116). Given their regulatory involvement in key biological processes, particularly EMT during the “inflammation to carcinoma” transformation, these miRNAs hold promise as potential biomarkers for GPL and GC.

**Figure 3:**
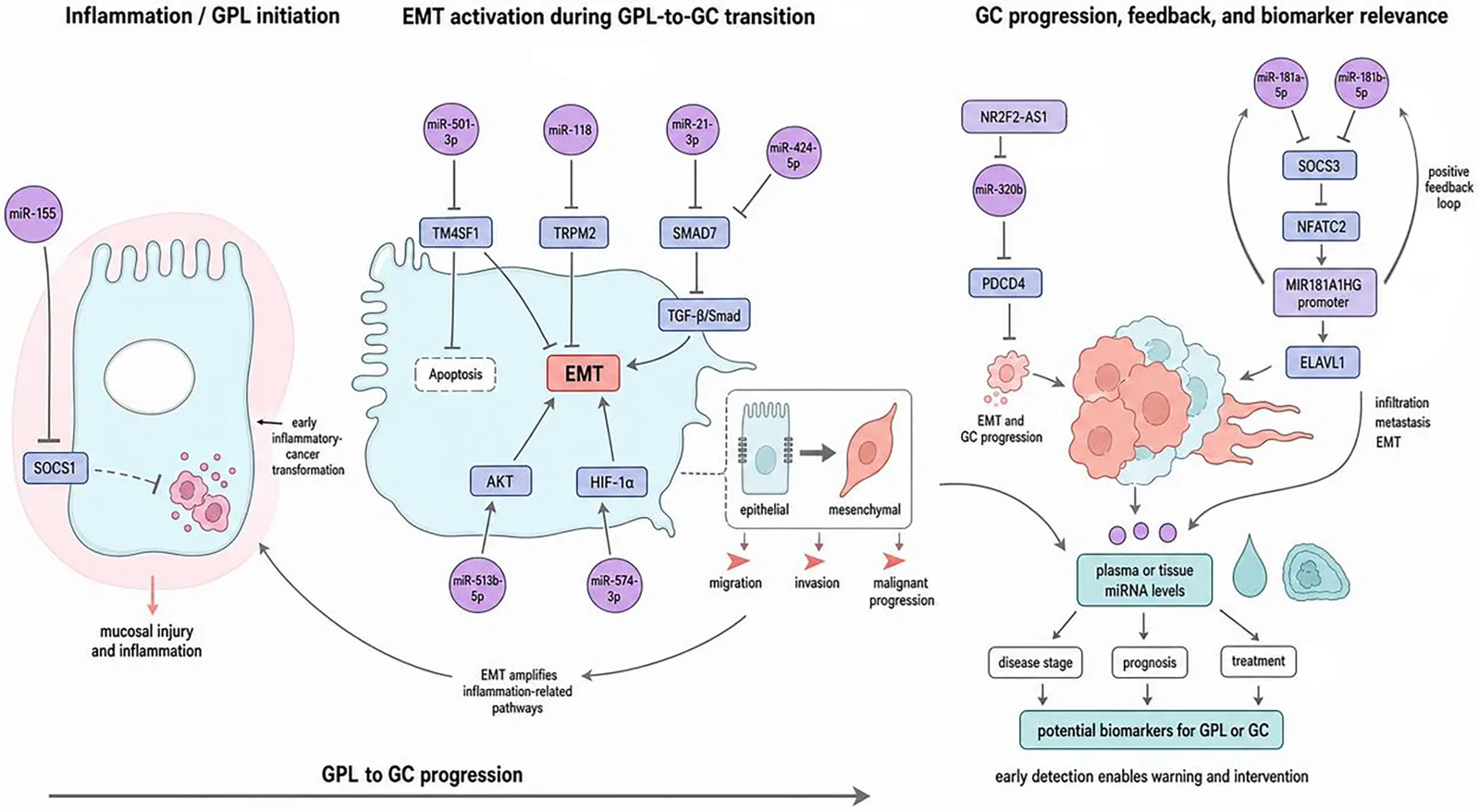
The regulatory mechanism of pro-EMT miRNAs in the transition from GPL to GC.

### 5.2 Types of anti-EMT miRNAs and their regulatory pathways

In contrast to oncogenic miRNAs, many tumor-suppressive miRNAs have been reported to be downregulated. Functional loss of these miRNAs frequently promotes EMT activation and the acquisition of malignant phenotypes (117–120) (**Figure 4**). The miR-200 family, which is recognized as a key suppressor of EMT in gastric epithelial cells, is markedly downregulated in both GPL and GC. This dysregulation activates the TGF-β and TNF-α signaling pathways, thereby contributing to TME formation and accelerating EMT progression (77). As a member of the miR-200 family, miR-200a additionally modulates the Bcl-2 signaling pathway, suppressing the proliferation, metastasis, and EMT of GC cells (121). Recent studies have identified that certain miRNAs, such as miR-520f-3p, are downregulated at early stages of GPL, mediating the KLF7/NFκB pathway, which induces inflammation and EMT in mouse gastric tissues. Conversely, overexpression of miR-520f-3p can counteract the effects of MNNG on migration, invasion, and EMT-related proteins in MC cells (122). The let-7 family, recognized as tumor-suppressive miRNAs, includes let-7i-3p, which targets CCND1 to inhibit the NFκB pathway, thereby playing a crucial role in suppressing EMT in GC (123). Similarly, let-7a-5p suppresses EMT in GC through the canonical Wnt/β-catenin signaling pathway (124). This pathway is frequently aberrantly activated by H. pylori infection or genetic mutations during gastric carcinogenesis, promoting cellular dedifferentiation and EMTT (98). In addition, miR-455-3p, miR-665, miR-497, and miR-203a-3p are downregulated in GC tissues or cells and inhibit EMT in gastric epithelial cells by modulating the Wnt/β-catenin signaling pathway (125–127). Notably, miR-203a-3p also suppresses the canonical TGF-β signaling pathway, which remains persistently activated in GPL and GC and drives epithelial cells toward a mesenchymal phenotype (97). miR-26-5p has likewise been reported to exert inhibitory effects on EMT (128). STAT3, a transcription factor frequently activated in GPL in response to chronic inflammation or cytokine stimulation, promotes EMT and the acquisition of stem-like properties (92, 129). MiRNAs can regulate STAT3 activity by modulating its expression or phosphorylation status (76). Furthermore, the PI3K/AKT/mTOR signaling pathway plays a critical role in the miRNA-mediated regulation of EMT in gastric epithelial cells. MiRNAs such as miR-30a-5p, miR-7, and miR-125a-5p can suppress this pathway, thereby inhibiting GC-induced EMT (130–132).

**Figure 4:**
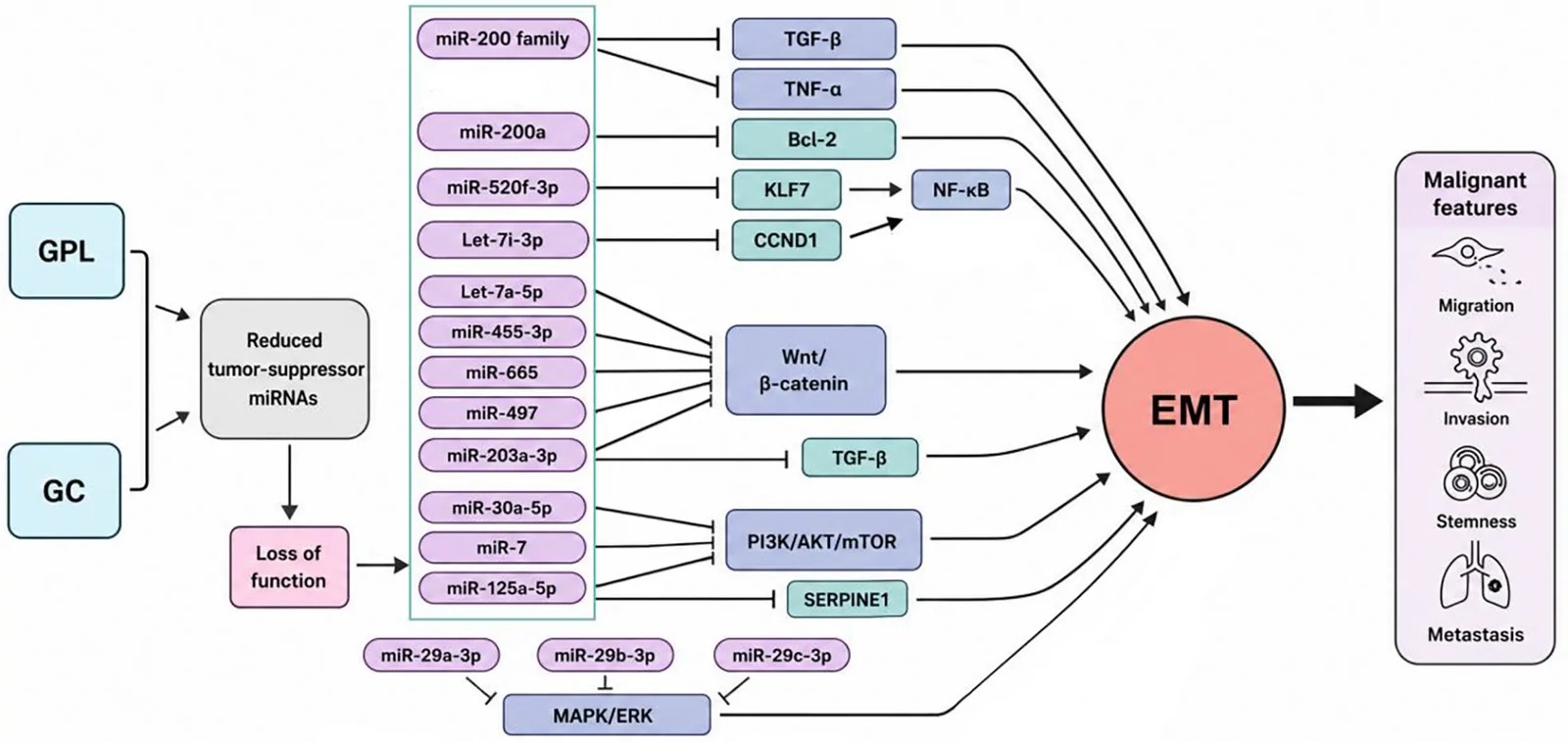
The regulatory framework of anti-EMT miRNA.

### 5.3 MiRNA regulatory networks modulating EMT

Recent studies indicate that miRNAs regulate EMT not merely through the modulation of individual signaling pathways, but rather via complex regulatory networks in which a single miRNA may target multiple pathways, whereas distinct miRNAs may converge on a common signaling axis (125–127). For example, the miR-29a/b/c-3p family has been reported to suppress EMT by modulating the MAPK/STAT3 signaling cascade (133). In addition, miR-29a-3p directly targets VAMP7, thereby inhibiting GC cell proliferation, migration, invasion, and EMT (18), while miR-29c attenuates EMT and tumor metastasis by regulating the VEGFA/VEGFR2/ERK signaling axis (134). Similarly, miR-125a-5p regulates EMT in GC by interacting with the PI3K/AKT pathway and targeting SERPINE1 (132, 135).

As a key branch of the MAPK signaling family, the ERK pathway constitutes an important component of the miRNA-mediated EMT regulatory network. Persistent ERK activation induced by RAS or growth factor stimulation promotes the expression of EMT-associated genes. Several miRNAs have been shown to inhibit this pathway, thereby restraining EMT in gastric epithelial cells (134). For instance, miR-490-3p targets HAVCR1 to modulate the MEK/ERK signaling pathway, inducing apoptosis while simultaneously suppressing cell proliferation, migration, and EMT (136). Likewise, miR-362-3p inhibits EMT through the DEP-1/ERK axis (137). MiR-145-5p further suppresses EMT by targeting SERPINE1 to inhibit ERK1/2 signaling and by interacting with YTHDF1 (138, 139). In addition, the Hippo signaling pathway, a vital regulator of cell proliferation and apoptosis, is also modulated during miRNA-mediated epithelial phenotypic reprogramming, thereby contributing to the progression of GC (140). Certain EMT-inhibitory miRNAs also modulate the stemness of GC cells. By targeting DDR2 and regulating the DDR2–mTOR–SOX2 axis, these miRNAs suppress the expression of stemness-associated markers, such as SOX2, thereby reducing GC cell stemness and inhibiting EMT programming. Collectively, these effects may impede the progression of gastric malignancies (141).

In miRNA-mediated regulation of EMT in gastric epithelial cells, individual miRNAs often target multiple downstream effectors. Representative examples include miR-195-5p (142, 143) and miR-493 (144, 145), whose expression and activity are regulated by distinct lncRNAs and circRNAs. Various ncRNAs act as competing endogenous RNAs or molecular sponges that sequester specific miRNAs, thereby attenuating miRNA-mediated repression of downstream EMT-associated signaling pathways and promoting the gastric malignant transformation (146–151). For example, lncRNA PCED1B-AS1 competitively binds miR-215-3p, leading to the upregulation of its downstream target, CXCR1, and consequently promoting EMT (152). Similarly, lncRNA FAM225A sequesters miR-206, thereby relieving miR-206-mediated repression of ADAM12. The resulting increase in ADAM12 expression reduces E-cadherin levels while enhancing Vimentin and N-cadherin expression, ultimately facilitating tumor growth (153). In addition, circKIF4A sequesters miR-144-3p and thereby modulates the expression of its downstream target EZH2 (154), whereas miR-144 has also been implicated in mTOR signaling pathway-driven EMT (155).

## 6 The prospect of miRNA-targeted therapy in GPL

MiRNAs exhibit remarkable stability and tissue- or lesion-specific expression patterns and can be readily detected in various biological fluids, including plasma, serum, and gastric juice. These properties support their potential as novel molecular tools for the early, non-invasive assessment and longitudinal monitoring of GPL. Recent advances in liquid biopsy research focusing on GPL and GC associated miRNAs have identified a series of circulating miRNA biomarkers that reflect gastric pathological alterations. These biomarkers show considerable promise for screening high-risk populations, predicting disease progression, and evaluating responses to endoscopic or pharmacological interventions (156). Furthermore, the integrchnology with advanced delivery systems may improve the precision and therapeutic efficacy of miRNA-based interventions, while potentially reducing the adverse effects associated with conventional chemotherapy (157) (**Table 3**).

**Table 3:** Utilization of MiRNAs in modulating EMT.

miRNAs	Source/strategies for intervention	Purpose	AUC	Sensitivity	Specificity	Reference
MiRNAs: non-invasive biomarkers for diagnostic and prognostic applications						
miR-1246、miR-21、miR-183-5p、miR-142-5p	small extracellular vesicle	Diagnostic、Monitoring	0.883	–	–	(191)
miR-129-1-3p	tissue、plasma	Diagnostic	0.681(CON vs CAG) 0.863(CON vs GC) 0.781(CAG vs GC)	–	–	(159)
miR-135b	exosomal from ascites	Diagnostic and Predictive	–	–	–	(162)
miR-140, miR-183, miR-30e, miR103a, miR-126, miR-93, miR-142, miR-21, miR-29c, miR-424 and miR-181a、miR-340	serum	Diagnostic	0.848	87.0%	68.4%	(166)
miR-145、miR-125b-2、miR-100、miR-221	memory B-cell-associated	Predictive	0.6303(OS at 1 years) 0.6403(OS at 3 years) 0.7092(OS at 5 years)	–	–	(165)
miR-18a、miR-181b、miR-335	serum	Diagnostic	0.86	–	–	(167)
miR-196a、miR-196b、miR-21	serum exosomes	Diagnostic	0.808	89%	88%	(192)
miR-196a-5p	tissue、plasma	Diagnostic	0.88(CON vs CAG) 0.931(CON vs GC) 0.663(CAG vs GC)	–	–	(159)
miR-196b、miR-221	serum	Diagnostic	0.954(GPL vs Healthy individuals) 0.9579(GPL vs GC)	–	–	(160)
miR-196b-3p、miR-92a-2-5p、miR-6791-3p	serum	Diagnostic	0.825(Discovery phase) 0.839(Training phase)	70%(Discovery phase) 73.3%(Training phase)	84.3%(Discovery phase) 91.4%(Training phase)	(158)
miR-21	serum exosomes	Diagnostic	0.89(GC) 0.74(GPL)	–	–	(161)
miR-30a-5p、miR-659-3p、miR-3917	tissue	Diagnostic and Predictive	0.87(Diagnostic) 0.82(Predictive)	–	–	(156)
miR-4257、miR-6785-5p、miR-187-5p、miR-5739	serum	Diagnostic	0.996	98.3%	97.7%	(168)
miR-5193	plasma	Predictive	0.801	–	–	(163)
miR-519a-3p	serum exosomes	Predictive	–	–	–	(164)
Extracellular vesicle therapy in modulating miRNA						
miR-144	PAMAM/miR-144 nanocarrier system	Intervention	–	–	–	(155)
miR-15b-5p	Exosomes circ0000549	Intervention	–	–	–	(178)
miR-196a-5p	Exosomes	Intervention	–	–	–	(16)
miR-204-5p	Tumor-Derived Exosomal LINC01480	Intervention	–	–	–	(179)
miR-29b	small extracellular vesicle	Intervention	–	–	–	(157)
miR-424-3p	Oxaliplatin-induced upregulation of exosomal miR-424-3p	Intervention	–	–	–	(180)
miR-486-5p	Exosomes	Intervention	–	–	–	(177)
Chemical interventions of the miRNA-driven EMT						
miR-200A	Xiaotan Sanjie decoction (XTSJ)	Intervention	–	–	–	(121)
miR-513b-5p	Jianpi Yangzheng decoction (JPYZ)	Intervention	–	–	–	(111)
miR-526b-5p	hesperetin	Intervention	–	–	–	(181)
miR-635	Asiaticoside	Intervention	–	–	–	(169)
miR-7	Baicalein (BAI)	Intervention	–	–	–	(131)

### 6.1 MiRNA as a non-invasive biomarker for risk assessment and prognostic prediction

Recent studies have identified distinct expression profiles of specific miRNAs across the healthy–premalignant–malignant continuum, highlighting their potential as biomarkers for risk prediction (158). Notably, miR-196a-5p and miR-129-1-3p exhibit dynamic and opposing expression patterns during disease progression: miR-196a-5p is progressively upregulated, whereas miR-129-1-3p is downregulated. Compared with healthy controls, miR-196a-5p achieved diagnostic sensitivities of 88.0% and 93.1% in patients with GPL and GC, respectively, while the corresponding sensitivities of miR-129-1-3p were 68.1% and 86.3%. The marked differences in the expression levels of these miRNAs among healthy, GPL, and GC populations support their potential utility as non-invasive biomarkers for the clinical diagnosis of GPL and GC (159). In addition, one study introduced a targeted, TMB-responsive bifunctional sensing platform utilizing miR-196b and miR-221 as indicators, which demonstrated commendable clinical applicability and accuracy when analyzing actual serum samples. This system also enables simultaneous dual-target detection, achieving areas under the curve (AUC) of 0.954 and 0.957 for combined target diagnostics, respectively (160).Another study developed a dual-signal amplification biosensor for the highly sensitive detection of exosomal miR-21, achieving diagnostic accuracies of 74% and 89% for GPL and GC, respectively (161). Furthermore, a pioneering electrochemiluminescence (ECL) sensor based on MoS2-QDs MXene heterostructures and Au NPs@biomimetic lipid layers was developed for the detection of exosomal miR-135b, facilitating the identification of exosomal miRNAs in the ascitic fluid of clinical GC patients and showcasing significant promise for exosomal diagnostics (162).

MiRNAs serve not only as biomarkers for disease diagnosis and risk assessment but also as promising tools for prognostic evaluation in patients with GC and GPL (163, 164). A recent single-cell RNA-sequencing study constructed an mRNA–miRNA co-expression network to identify miRNAs associated with memory B cells. Based on these miRNAs, a prognostic risk model was developed and validated, achieving time-dependent AUC values of 0.6303, 0.6403, and 0.7092 for predicting 1-, 3-, and 5-year overall survival in patients with GC, respectively. These findings provide new insights into prognostic assessment and risk stratification in this patient population (165).

A growing body of evidence has explored the incorporation of multi-miRNA signatures into diagnostic frameworks for GPL and GC (166). In one study, comprehensive analysis of serum samples from 598 patients with GC identified a three-miRNA panel comprising miR-18a, miR-181b, and miR-335. This panel exhibited robust diagnostic performance across all disease stages, with an AUC of 0.86 (95% CI: 0.83-0.90) (167). In another study, high-sensitivity DNA microarray analysis of 2,565 miRNA sequences in a cohort of 1,417 patients with GC established a four-miRNA panel consisting of miR-4257, miR-6785-5p, miR-187-5p, and miR-5739, which achieved a sensitivity of 98.3% and a specificity of 97.7% (168).

### 6.2 Extracellular vesicle therapy in regulating miRNA and the EMT pathway

The intervention strategy focused on the miRNA EMT axis has demonstrated significant potential in the reversal therapy for GPL (143, 169). Multiple miRNAs are markedly downregulated in both GPL and GC, whereas exosome-mediated miRNA delivery has demonstrated efficacy in suppressing EMT (170–175). For instance, the delivery of miR-200a via extracellular vesicles derived from mesenchymal stem cells substantially increases miR-200a levels in GC cells, thereby upregulating E-cadherin and downregulating β-catenin, vimentin, ZEB1, and Snail1, ultimately inhibiting EMT (176). In addition, a novel nanocarrier system protects miR-144 from nuclease-mediated degradation and enables its stable delivery to GC cells. This approach reduces mTOR protein expression, inhibits EMT, and suppresses tumor growth, thus representing a promising therapeutic strategy for GC (155). Recent evidence further indicates that various miRNAs carried by GC-associated extracellular vesicles play pivotal roles in regulating EMT and contribute to GC progression and peritoneal metastasis (16, 177). Exosomal delivery of upstream regulatory non-coding RNAs can also indirectly modulate miRNA activity, thereby suppressing EMT and restraining the gastric malignant progression (178, 179). Certain pharmacological agents possess the capability to modulate the targeting of miRNA within extracellular vesicles, influencing the EMT. For instance, Oxaliplatin has been demonstrated to elevate the expression levels of miR-424-3p in extracellular vesicles derived from human bone marrow mesenchymal stem cells (hBM MSCs), subsequently targeting and downregulating RHOXF2 expression, which ultimately impedes the EMT process in GC cells (180).

### 6.3 Targeting the miRNA EMT pathway through chemical interventions

Numerous studies have demonstrated that natural compounds can reverse EMT by modulating the expression of specific miRNAs, thereby suppressing disease progression. Asiaticoside induces endoplasmic reticulum stress through the miR-635/HMGA1 signaling axis, resulting in the downregulation of the mesenchymal marker N-cadherin and the concomitant upregulation of the epithelial marker E-cadherin, ultimately inhibiting GC progression (169). Hesperetin have been found to inhibit the sponge like effect of extracellular vesicle circ008274 on miR-526b-5p, suppress the proliferation, EMT, and cancer stem like characteristics of TGES-1 cells (MNNG -induced malignantly transformed GES-1 cells) (181). Astragaloside IV (AS-IV) has also been shown to inhibit EMT and angiogenesis in GC by upregulating miR-195-5p (143). Additionally, Baicalin (BAI) influences focal adhesion kinase (FAK) through the PI3K/AKT signaling pathway, impacting the proliferation, metastasis, EMT, and angiogenesis of GC cells (131). Traditional Chinese medicine (TCM) formulations also exhibit considerable therapeutic potential by regulating miRNA-associated EMT signaling pathways. For example, Xiaotan Sanjie Decoction (XTSJ) dose-dependently downregulates lncRNA ATB while upregulating miR-200a, thereby modulating EMT- and Bcl-2-related signaling and inhibiting GC proliferation and metastasis (121). Studies have shown that Jianpi Yangzheng decoction (JPYZ) can reduce miR-513b-5p expression, inhibit the AKT/mTOR signaling pathway, and holds substantial therapeutic value in curbing GC progression. Collectively, these findings substantiate the regulatory effects of bioactive TCM components on the miRNA–EMT axis and provide a scientific basis for developing safe and effective chemopreventive agents (111).

In conclusion, elucidating the roles of miRNAs in the initiation and progression of GPL and its malignant transformation into GC is of considerable clinical significance. Interdisciplinary collaboration and investigation will further advance research on the “inflammation to carcinoma” transformation in GPL, thereby providing renewed hope for affected patients.

## 7 Discussion

The present review provides a comprehensive overview of the miRNA-mediated regulatory networks governing EMT during the progression from chronic gastritis to GPL and ultimately to GC (**Figure 5**). By integrating evidence from diverse experimental models, we have delineated how inflammation-driven miRNA dysregulation orchestrates a complex signaling landscape that promotes epithelial plasticity, stemness acquisition, and malignant transformation. Nevertheless, several critical limitations of the current body of evidence must be acknowledged, and important transformation barriers must be addressed before miRNA-based strategies can be realized in clinical practice.

**Figure 5:**
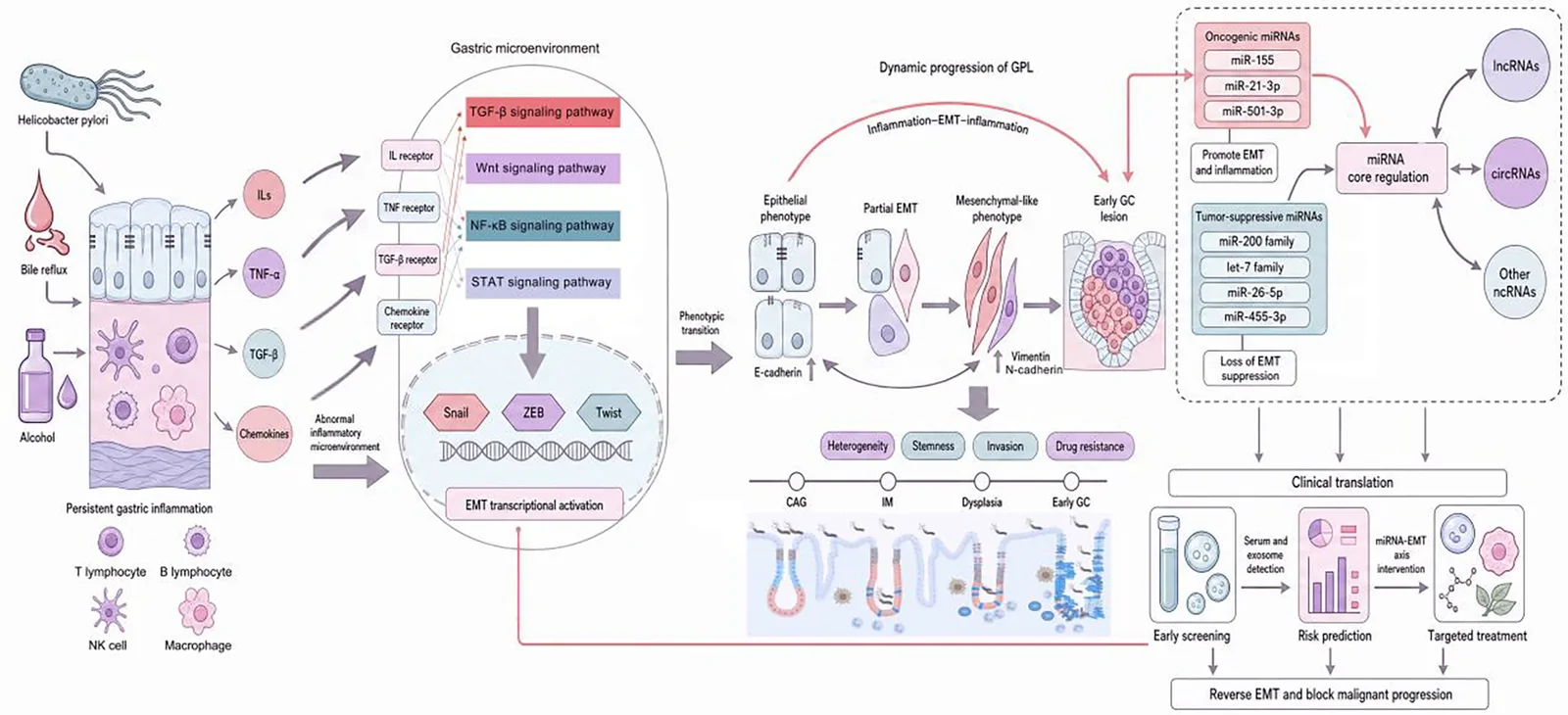
The involvement and potential applications of inflammation, EMT, and miRNAs in the progression from GPL to GC.

First, current evidence regarding the role of the miRNA–EMT axis in gastric malignant transformation has largely been derived from heterogeneous biological models, including animal models of gastritis, GPL, and GC, as well as cellular models such as MC cells and GC cell lines (74, 182–185). Available findings suggest that miRNA-mediated regulation of EMT varies across disease stages, with pEMT predominating during the early stages and EMT becoming progressively more pronounced during later stages (186). However, dynamic studies systematically characterizing the regulatory effects of the miRNA–EMT axis throughout the entire gastritis–GPL–GC cascade remain lacking (187). Future studies should leverage advanced approaches, including single-cell profiling, organoid models, longitudinal human cohorts with serial biopsy sampling, and genetically engineered mouse models that recapitulate the relevant pathological cascade, to dynamically elucidate the role of miRNAs in EMT regulation throughout gastric malignant progression. Such studies may establish a causal relationship between miRNA-mediated EMT and progressive gastric carcinogenesis, thereby providing promising strategies for the prevention and treatment of GPLs and GC.

Secondly, the clinical application of circulating miRNAs as non-invasive biomarkers for GPL and GC remains limited by several factors. (a) Standardization: No universally accepted protocols have been established for the collection, preparation, extraction, and normalization of circulating miRNAs. Substantial variability persists in sample type (serum versus plasma), blood collection procedures, pre-analytical processing, and the selection of reference controls for data normalization (188). (b) Specificity: Some miRNA signatures associated with GPL and GC overlap with those identified in other gastrointestinal malignancies, such as colorectal cancer (189), and even in systemic disorders, including autoimmune diseases and metabolic syndrome (190). This overlap raises concerns regarding their disease specificity. (c) Gap between discovery and validation: Most studies investigating miRNA biomarkers have been retrospective, single-center studies with relatively small sample sizes. Prospective validation in large, multicenter clinical cohorts remains lacking (191, 192), as do long-term follow-up data and comprehensive assessments of clinical utility.

Third, targeting the miRNA–EMT axis holds considerable therapeutic potential but is also associated with substantial safety and delivery challenges. (a) Off-target effects: miRNAs exert their regulatory functions by binding to the 3′ untranslated regions (3′UTRs) of hundreds of potential target mRNAs. Consequently, therapeutic modulation of a single miRNA—such as antagomir-mediated silencing of oncogenic miR-21 or restoration of the tumor-suppressive miR-34a—may inadvertently alter the expression of unintended genes involved in essential cellular processes (193), thereby causing unpredictable toxicity. Given the pleiotropic nature of miRNA-mediated regulation, comprehensive off-target profiling is required before clinical translation. (b) Delivery challenges: Efficient *in vivo* delivery of miRNA-based therapeutics to gastric epithelial cells remains a major obstacle. Unprotected miRNA molecules are rapidly degraded by RNases in the gastrointestinal tract and systemic circulation. Although nanoparticle- and exosome-based delivery systems have demonstrated promise in preclinical models, critical issues—including biodistribution, cellular uptake efficiency, endosomal escape, immune activation, and long-term biocompatibility—must be rigorously addressed (194).

Fourth, the clinical translation of miRNA-based diagnostic and therapeutic strategies remains challenging. To date, no miRNA-based drugs have been approved for the treatment of gastrointestinal tumors, largely because manufacturing standards, dosing regimens, pharmacokinetic and pharmacodynamic profiles, and cumulative toxicity require extensive validation before clinical implementation.

Based on the current state of the field, future research should focus on: (a) standardizing protocols for circulating miRNA detection; (b) prospectively validating potential miRNA biomarkers in large, multicenter cohorts; (c) elucidating the molecular mechanisms underlying the miRNA–EMT axis within the complete gastric microenvironment by integrating patient-derived organoids, spatial transcriptomics, longitudinal human cohort studies with serial biopsy sampling, and genetically engineered mouse models that recapitulate the relevant signaling cascades; (d) developing safe drug-delivery systems capable of precisely targeting gastric epithelial cells.

## 8 Conclusion

In conclusion, chronic gastric inflammation establishes a persistent pro-inflammatory microenvironment that activates multiple canonical signaling pathways, thereby driving the dysregulated expression of EMT-TFs and promoting the acquisition of mesenchymal-like features by gastric epithelial cells. GPL progression is accompanied by dynamic pEMT and the emergence of hybrid E/M phenotype, which endows gastric epithelial cells with enhanced stemness, invasiveness, and resistance to therapeutic interventions, ultimately accelerating the transition from chronic inflammation to GC.

MiRNAs serve as central regulators with dual roles in the pathological processes described above. From a clinical translational perspective, their high tissue specificity and stability in body fluids make them promising non-invasive biomarkers for the early screening, risk prediction, and prognostic assessment of GPL and GC. Meanwhile, targeted modulation of the miRNA–EMT axis, through strategies such as extracellular vesicle-mediated miRNA delivery and the regulation of miRNA expression by bioactive compounds derived from traditional Chinese medicine, has demonstrated considerable potential for reversing EMT and preventing the malignant progression of GPL.

However, before these strategies can be translated into clinical practice, several major translational barriers must be rigorously addressed, including insufficient standardization, limited specificity, the gap between discovery and validation, suboptimal targeting, and low delivery efficiency. Through such rigorous, multidisciplinary efforts can miRNA-based strategies for the early detection, risk stratification, and targeted intervention of GPL may be translated from promising concepts into clinically actionable tools.
