# Supplementary material for: From inflammation to carcinogenesis: MiRNA regulates epithelial-mesenchymal transition to facilitate malignant progression of gastric precancerous lesions
Source: Front Oncol. 2026 Sep 28;16:1959240. doi: 10.3389/fonc.2026.1959240 (PMC13655720; doi:10.3389/fonc.2026.1959240)
Supplement: Supplementary file 1 [file Table1.docx]

Supplementary Material

# 1 Method

This study used a narrative review method to analyze the literature on miRNA regulation of EMT during the transition from GPL to GC from 2007 to 2026, and summarized the regulation process and application prospects of miRNA on EMT.

**Table S1.** Search strategy summary

| Items | Specification |
| --- | --- |
| Date of search | 2026.02.08–2026.07.07 |
| Databases and other sources searched | PubMed and Web of Science |
| Search terms | ("MicroRNAs" OR miRNA OR microRNA) AND  ("Intestinal Metaplasia" OR "Gastritis, Atrophic" OR "Dysplasia" OR "gastric precancerous lesions" OR "precancerous stomach") AND  ("Inflammation" AND "Carcinogenesis" OR "inflammation-related carcinogenesis" OR "inflammation-driven carcinogenesis") AND  ("Epithelial-Mesenchymal Transition" OR "EMT regulation" OR EMT) |
| Timeframe | 2007–2026 |
| Inclusion and exclusion criteria | Inclusion criteria: all kinds of studies |
|  | Exclusion criteria: studies published in languages other than English |
| Selection process | All the authors conducted the search and contributed to the final version of the manuscript |
